# Intermediate Visual Performance of Clareon Versus Eyhance Enhanced Monofocal Intraocular Lenses

**DOI:** 10.3390/diagnostics16132011

**Published:** 2026-06-27

**Authors:** Marlena Cwynar-Ptak, Wiktoria Czuj-Porębska, Aleksandra Prus-Ludwig, Jarosław Piłat, Dariusz Dobrowolski, Edward Wylęgała, Bogumił Wowra

**Affiliations:** 1 Chair and Department of Ophthalmology, Faculty of Medical Sciences in Zabrze, Medical University of Silesia, 40-760 Katowice, Poland; 2 Department of Ophthalmology, Saint Barbara Hospital, Trauma Center, 41-200 Sosnowiec, Poland; 3 Department of Ophthalmology, District Railway Hospital in Katowice, Medical University of Silesia, 40-760 Katowice, Poland

**Keywords:** enhanced monofocal intraocular lenses, Clareon IOL, TECNIS Eyhance IOL, intermediate visual acuity, cataract surgery

## Abstract

**Background/Objectives:** Intermediate vision has become increasingly important after cataract surgery because many daily activities require functional visual performance beyond distance vision alone. Enhanced monofocal intraocular lenses may improve intermediate visual function while maintaining good distance visual acuity. The aim of this study was to compare binocular distance and intermediate visual outcomes after bilateral implantation of Clareon and TECNIS Eyhance intraocular lenses. **Methods:** This was a single-center, non-randomized comparative observational study with assessment of postoperative visual outcomes. Eighty-six patients who had previously undergone uncomplicated bilateral age-related cataract surgery with implantation of the same IOL model in both eyes were included. Forty-two patients received Clareon IOLs and forty-four patients received TECNIS Eyhance IOLs. Postoperative assessment was performed at least 12 weeks after surgery of the second eye. Binocular corrected distance visual acuity (CDVA) and distance-corrected intermediate visual acuity (DCIVA) were measured using ETDRS charts at 4 m and 66 cm. **Results:** Baseline biometric and clinical parameters were comparable between groups. Mean postoperative DCIVA was 0.23 ± 0.09 logMAR in the Clareon group and 0.21 ± 0.08 logMAR in the Eyhance group. The mean between-group difference, calculated as Clareon minus Eyhance, was 0.02 logMAR, with a 95% confidence interval from −0.02 to 0.06 logMAR. Mean binocular CDVA was 0.02 ± 0.02 logMAR in the Clareon group and 0.02 ± 0.03 logMAR in the Eyhance group, with a 95% confidence interval for the between-group difference from −0.01 to 0.01 logMAR. Mean postoperative manifest refraction spherical equivalent was −0.27 ± 0.42 D in the Clareon group and −0.29 ± 0.37 D in the Eyhance group. **Conclusions:** Both Clareon and TECNIS Eyhance IOLs provided good binocular distance and intermediate visual acuity after bilateral implantation. Intermediate visual performance after Clareon implantation was comparable to that achieved with TECNIS Eyhance, while distance visual acuity remained similarly high in both groups. These findings suggest that both IOL models may represent useful options for patients undergoing cataract surgery who expect good distance vision and functional intermediate visual performance.

## 1. Introduction

The ocular lens is a small, transparent structure placed in the anterior segment of the eye. Its main function is to focus the light on the retina and allow the eye to see objects at different distances. Opacification of the lens is known as a cataract, and in most cases, it is age-related. Cataracts commonly lead to reduced visual acuity, decreased contrast sensitivity, and deterioration of overall visual function, thereby limiting daily activities and independence [1]. Due to cataracts, in 2020, approximately 94 million patients aged 50 and older worldwide experienced visual impairment or vision loss, which may negatively influence independence, mobility, social activity, and overall quality of life, particularly in older adults. As a result of population aging, this number is expected to increase further [2]. Cataracts are considered one of the leading causes of reversible visual impairment worldwide. The constantly growing number of affected individuals is not only a medical challenge but also an important social and economic burden.

The pathophysiology of cataracts is not fully understood. Moreover, there is no effective pharmacological treatment for cataracts. Therefore, surgery remains the only effective method to treat the condition and restore visual acuity and function [1,2]. The procedure involves the removal of the opacified lens and implantation of an artificial intraocular lens (IOL). The most common lens type used during cataract surgery is a monofocal IOL, which is designed to provide clear vision at a single distance based on the selected refractive target. Consequently, patients implanted with this type of IOL often require spectacles for good intermediate and near vision [3].

Higher levels of spectacle-independence may be achieved with premium IOLs, e.g., trifocal IOLs and extended depth of focus IOLs (EDOF). EDOF lenses provide improved distance and intermediate vision, whereas trifocal lenses offer additional near visual function. However, these lenses may be associated with higher costs for patients. Moreover, they may be associated with undesirable photic phenomena [4,5]. Although multifocal and EDOF technologies may reduce postoperative spectacle dependence, not all patients should be qualified for these IOLs. Multifocal IOLs are often discouraged in patients with retinal disorders or an increased risk of retinal disease, such as diabetic retinopathy, age-related macular degeneration, or epiretinal membrane, because these conditions may further compromise contrast sensitivity and visual quality [6]. Moreover, some individuals may prioritize preservation of contrast sensitivity and minimization of photic phenomena over complete spectacle independence. In such cases, enhanced monofocal IOLs may represent an alternative approach aimed at extending the functional range of vision.

The success of the surgical treatment is no longer defined only by medical criteria. A major determinant of treatment effectiveness is patient-reported quality of life (QoL) after surgery. Advances in cataract surgery, as well as IOL technology, have brought significant benefits in terms of improving patient QoL [7]. In addition, Xuepei Li et al. demonstrated an improvement in psychological distress after cataract surgery, together with improved visual acuity [8]. Therefore, modern cataract surgery should no longer be considered only a vision-restoring medical procedure but also an optical intervention aimed at improving patient satisfaction. Consequently, increasing attention has been focused on improving visual performance at multiple distances while preserving overall quality of vision.

In recent years, there has been increasing recognition of the importance of intermediate vision. Many everyday activities, including computer work, tablet or smartphone use, cooking, shopping, driving and playing musical instruments, require intermediate vision. Currently, many individuals over the age of 60 engage in professional, social and digital activities that require clear intermediate vision. Hence, spectacle-independent intermediate vision may soon become an expected outcome [9,10]. Activities requiring intermediate vision currently constitute a major component of everyday life in both professionally active and retired individuals. Therefore, postoperative visual expectations may soon extend beyond distance vision alone and include comfortable visual performance at intermediate distances in daily activities. It has been shown that freedom from spectacles can significantly improve QoL [11].

In response to these growing expectations, the optical design of conventional monofocal IOLs has evolved to enhance functional vision at intermediate distances while maintaining distance visual acuity [12,13]. Unlike EDOF and diffractive multifocal IOLs, new-generation IOLs broaden the range of vision by modifications of the optical surface profile [14]. Initially, aspheric IOLs were introduced to minimize the impact of corneal spherical aberrations, which are commonly associated with traditional IOLs, on postoperative vision. However, a small amount of positive spherical aberration is used in the refinement of the anterior optic surface to extend the longitudinal range of focus in enhanced monofocal IOLs [15]. This approach might improve intermediate visual performance while maintaining distance visual acuity and contrast sensitivity, with a low incidence of photic phenomena [16].

The aim of this study was to compare visual outcomes in patients with bilateral implantation of two enhanced IOLs: Clareon (Alcon Vision, LLC., Fort Worth, TX, USA) and Eyhance (Johnson & Johnson Vision, Jacksonville, FL, USA).

## 2. Materials and Methods

### 2.1. Study Design

This was a single-center, retrospective, non-randomized comparative observational study designed to assess postoperative visual performance following bilateral cataract surgery with implantation of either Clareon or TECNIS Eyhance intraocular lenses. The analysis focused on binocular distance and intermediate visual acuity outcomes in a group of patients who had undergone routine cataract surgery with bilateral implantation of the same IOL model.

The study was conducted in accordance with the principles of the Declaration of Helsinki. Due to the retrospective observational design and the use of anonymized clinical data obtained during routine medical care, separate institutional ethics committee approval was not required. All patients had previously provided written informed consent for cataract surgery and consented to the anonymous use of clinical data for scientific purposes at the time of hospital admission.

### 2.2. Study Population

The primary inclusion criterion for the study was previous uncomplicated age-related cataract surgery with implantation of the same model of either the Clareon monofocal IOL (Alcon Vision, LLC.) or the TECNIS Eyhance ICB00 IOL (Johnson & Johnson Vision) in both eyes. All surgeries were carried out at the Department of Ophthalmology, Railway Hospital Katowice, Medical University of Silesia. The study required a minimum postoperative follow-up of three months.

Additional inclusion criteria were transparent ocular media, absence of clinically significant posterior capsular opacification and a postoperative corrected distance visual acuity (CDVA) of 0.1 logMAR (20/25) or better in each eye. In cases where the yttrium aluminum garnet (YAG) laser capsulotomy was performed, the required time from the procedure to postoperative assessment was at least two weeks.

Patients with a medical history of ocular or systemic disease that could interfere with visual outcomes, including glaucoma, corneal dystrophies or irregularities, strabismus, retinal or optic nerve pathology, preexisting conditions that may affect cataract removal (such as pseudoexfoliation syndrome) or any clinically significant acute or chronic illness were excluded from the study. A history of previous anterior segment surgery other than cataract extraction, posterior segment surgery, or any intraoperative complication during cataract surgery also led to exclusion from the analysis.

### 2.3. Intraocular Lenses

Two enhanced monofocal IOL models were evaluated in this study.

The Clareon monofocal IOL, introduced by Alcon Laboratories, Inc. (Fort Worth, TX, USA), is a one-piece, hydrophobic acrylic lens with an aspheric anterior surface and spherical posterior surface. The Clareon CCA0T0 model, which provides ultraviolet light filtration, and the Clareon CNA0T0 model, which additionally incorporates a blue light-filtering chromophore, share the same optical design. Hence, they were both included in the study [17].

The TECNIS Eyhance ICB00 (Johnson & Johnson Vision) is likewise a single-piece, hydrophobic acrylic monofocal intraocular lens with a 6.0 mm optic and a total length of 13.0 mm. Its central anterior optic surface, within the innermost 2.0 mm diameter, is aspheric and incorporates a gradual increase in central optical power, which aims to slightly extend the depth of focus [18].

### 2.4. Surgical Technique

Standard phacoemulsification was performed by experienced surgeons in all patients. In each case, the selected IOL was implanted into the capsular bag. The refractive target and IOL type were selected individually based on preoperative examination, consultation with the patient and consideration of preoperative refraction. The second eye was operated on after adequate recovery of the first eye, with implantation of the same type of intraocular lens.

### 2.5. Postoperative Examination

Postoperative assessment was performed at a minimum of 12 weeks after the surgery of the second eye. Binocular corrected distance visual acuity and distance-corrected intermediate visual acuity were measured using ETDRS charts. Intermediate visual acuity was evaluated at 66 cm, whereas distance visual acuity was assessed at a distance of 4 m. All visual acuity measurements were conducted with distance correction in place to reduce the risk of potential interference of residual refractive error with visual outcomes.

### 2.6. Outcome Measures

The primary endpoint was binocular distance-corrected intermediate visual acuity (DCIVA) in both groups. Secondary outcome measures included corrected distance visual acuity (CDVA), postoperative manifest refraction spherical equivalent (MRSE), and postoperative residual astigmatism (PRA).

All statistical analyses were performed using TIBCO Statistica, version 13.3.1 (TIBCO Software Inc., Palo Alto, CA, USA). No imputation was applied for missing data. Data were assumed to be approximately normally distributed when skewness values were within ±2 and kurtosis values were within ±7. The Welch Two-Sample t-test was used for parametric data, whereas non-parametric variables were analyzed using the Wilcoxon rank-sum test. For all statistical analyses, a *p*-value of less than 0.05 was considered statistically significant.

## 3. Results

Eighty-six patients were analyzed after meeting the inclusion criteria. Each participant underwent implantation of the same selected IOL model in both eyes. The Clareon IOLs were implanted in 42 patients, whereas TECNIS Eyhance IOLs were implanted in 44 patients. Baseline clinical and ocular parameters were generally comparable between groups, as shown in Table 1. 

Biometric characteristics assessed before surgery, including anterior chamber depth, axial length, pupil size, corneal astigmatism, average keratometry, and IOL power, did not show clinically relevant differences between the two groups. The mean age was 67.56 ± 6.61 years in the Clareon group and slightly lower in the Eyhance group, at 64.46 ± 6.61 years. Female patients constituted the majority in both study groups, accounting for 57.1% of the Clareon group and 54.5% of the Eyhance group. None of the patients meeting the inclusion criteria were qualified for Toric IOL implantation in this study.

Refractive outcomes after cataract surgery were close to the intended mild myopic target in both groups. Mean postoperative manifest refraction spherical equivalent (MRSE) was −0.27 ± 0.42 D in the Clareon group and −0.29 ± 0.37 D in the Eyhance group. Mean postoperative residual astigmatism (PRA) was 0.46 ± 0.52 D in the Clareon group and 0.48 ± 0.42 D in the Eyhance group.

Binocular visual acuity outcomes are summarized in Table 2. Distance-corrected intermediate visual acuity (DCIVA) showed comparable results in both IOL groups. The Clareon group achieved a mean binocular DCIVA of 0.23 ± 0.09 logMAR, while the Eyhance group achieved a mean binocular DCIVA of 0.21 ± 0.08 logMAR. The mean difference between groups was 0.02 logMAR. This corresponds to approximately one ETDRS letter. The 95% confidence interval for this difference ranged from −0.02 to 0.06 logMAR. The observed difference remained within the predefined non-inferiority margin of 0.10 logMAR.

Corrected distance visual acuity remained high in both groups, and patients reported high postoperative satisfaction. Mean binocular CDVA was 0.02 ± 0.02 logMAR in the Clareon group and 0.02 ± 0.03 logMAR in the Eyhance group. The 95% confidence interval for the between-group difference ranged from −0.01 to 0.01 logMAR. As with DCIVA, this result remained within the predefined non-inferiority margin, indicating no clinically relevant difference in distance visual acuity between the two types of IOLs.

The visual acuity results indicate that both IOL models provided stable binocular visual performance after implantation of the same IOL in both eyes. The small difference observed in DCIVA did not translate into clinically meaningful differences between groups. Distance visual acuity remained similarly high in both groups. These findings were consistent across the main postoperative visual endpoints evaluated in this study.

## 4. Discussion

The main purpose of this study was to compare the intermediate visual outcomes of the Clareon and Eyhance monofocal IOLs. The key finding of this study was that binocular intermediate visual acuity after Clareon implantation was not clinically inferior to that achieved with TECNIS Eyhance, while distance visual acuity remained similarly high in both groups. This result is consistent with the findings reported by J. Morgan Micheletti et al. [19].

The difference in mean DCIVA between groups was 0.02 logMAR, corresponding to approximately one ETDRS letter. Such a small difference is unlikely to be meaningful in daily visual function. In studies comparing IOL outcomes, statistical differences may not be clinically meaningful for patients. In the present study, the between-group difference in DCIVA remained below the predefined non-inferiority threshold.

From a clinical perspective, this suggests that the intermediate visual performance of Clareon and Eyhance was highly comparable in this study population. Therefore, although the Eyhance IOL is designed specifically to enhance intermediate vision, the Clareon IOL also provides functional intermediate visual acuity without compromising distance visual outcomes.

The mean DCIVA values observed in this study, 0.23 logMAR for the Clareon group and 0.21 logMAR for the Eyhance group, as well as the mean CDVA value of 0.02 logMAR in both groups, are consistent with previously published results [13,18,20,21]. Together with recent findings indicating that enhanced monofocal IOLs may improve intermediate visual acuity while maintaining good distance vision and contrast sensitivity, these results further support the stable and clinically useful visual performance of this IOL category [22,23,24]. Similar improvements have also been reported in patients with Fuchs endothelial corneal dystrophy. This observation may be relevant because coexisting corneal pathology, such as Fuchs endothelial corneal dystrophy, can reduce visual acuity and contrast sensitivity, making postoperative visual outcomes less predictable after cataract surgery. The favorable outcomes reported with enhanced monofocal IOLs suggest that extending intermediate vision does not necessarily compromise overall visual function in these groups of patients.

Moreover, bilateral implantation of the enhanced monofocal IOL has been shown to have no adverse effect on patient-reported quality of life [25]. Previous studies comparing Eyhance IOL with conventional monofocal IOLs consistently demonstrated improved intermediate visual acuity without deterioration of distance vision or contrast sensitivity [26]. Additionally, comparative studies of different enhanced monofocal IOL models have also shown that this lens category may provide excellent intermediate visual outcomes while maintaining good distance visual acuity [27]. These observations are clinically relevant because intermediate visual tasks constitute an increasingly important component of postoperative patient expectations. The findings of the present study are consistent with this trend [22]. Long-term stability of visual performance with Eyhance IOLs was recently supported by a 5-year follow-up study by Mencucci et al. [9]. The authors reported maintained distance and intermediate visual acuity at a stable level over time, indicating that the optical performance of enhanced monofocal IOLs remains stable during long-term postoperative observation [9]. This may be particularly relevant in an aging population. The postoperative intermediate visual acuity observed in the Clareon group also supports recent findings showing that patients implanted with this type of IOL reported spectacle independence for intermediate tasks [15]. Reduction in spectacle dependence may have an important influence on postoperative satisfaction and quality of life in all age groups of patients, including adults who more frequently engage in activities requiring intermediate vision in everyday settings.

From both a clinical and everyday-life perspective, these findings are relevant because many patients expect useful vision not only at distance, but also at intermediate working distances after cataract surgery. This expectation is becoming more important as older adults want to remain professionally, socially, and digitally active for longer periods of life. Therefore, the demand for IOLs that provide functional intermediate and distance visual acuity is likely to increase.

Monofocal IOLs remain the most common type of IOLs implanted during cataract surgery. In certain monofocal IOL designs, particularly spherical or aberration-neutral models, limited intermediate vision has been achieved due to residual spherical aberration and an increased depth of focus [23,28].

The TECNIS Eyhance IOL was developed to improve intermediate visual performance while maintaining the characteristics of a monofocal IOL by modifying the anterior surface. Therefore, it is an appropriate reference lens in this comparison. In the present study, the Eyhance group achieved good binocular DCIVA and excellent CDVA, which is consistent with its intended optical profile. The comparable outcomes observed in the Clareon group therefore suggest that Clareon may also provide clinically useful intermediate vision.

Clareon is classified as a monofocal IOL and provides excellent distance vision. However, its optical design may also support a certain degree of intermediate visual function. The lens incorporates an aspheric anterior surface and spherical posterior surface, which may contribute to extending the functional range of focus [17].

Similar conclusions have been reported for the AcrySof IQ SN60WF monofocal IOL, which shares several optical characteristics with the Clareon model. Previous studies evaluating this optical platform demonstrated preservation of functional intermediate vision despite classification as a monofocal IOL [13]. Similar findings were reported for the toric Clareon IOL [29]. These findings suggest that comparatively small modifications in optical surface geometry may cause changes in the functional range of vision achieved after cataract surgery. These findings may also help explain the preserved intermediate vision observed in the Clareon group in the present study.

This study has several limitations. First, it was a single-center study with a limited sample size, and the study was not randomized. The choice of IOL type was individualized during preoperative consultation, which may have introduced selection bias. Second, no Toric versions of the Eyhance IOL or Clareon IOL were included in this study. Therefore, the results should not be directly extrapolated to patients requiring Toric correction. In addition, patients who were not targeted for emmetropia were included in this study. However, visual acuity was measured with distance correction to minimize refractive bias and reduce the influence of residual postoperative refractive error. Another limitation was the lack of standardized questionnaires to assess spectacle independence, dysphotopsia, patient satisfaction, and vision-related quality of life. Contrast sensitivity and photic phenomena were assessed clinically; however, standardized quantitative assessment methods were not used. Finally, the follow-up period was limited to the early postoperative period; therefore, longer observation would be necessary to confirm the stability of the results over time.

From a healthcare-system perspective, cataract surgery is a cost-effective procedure, as it provides almost immediate improvement in visual function and QoL [30]. From a practical perspective, enhanced monofocal IOLs may represent a clinically useful option for patients seeking improved intermediate vision without the potential disadvantages associated with multifocal technologies. This may be particularly relevant for patients who prioritize high-quality distance vision but also wish to maintain functional vision for everyday intermediate tasks. In routine cataract surgery, such lenses may help bridge the gap between standard monofocal IOLs and more advanced presbyopia-correcting technologies. At the same time, the lower cost of enhanced monofocal IOLs compared to premium presbyopia-correcting IOLs may facilitate their accessibility in routine cataract surgery and support broader postoperative visual rehabilitation. These considerations may be important during preoperative counseling and shared decision-making regarding IOL selection.

Overall, both Clareon and Eyhance monofocal IOLs provided comparable distance and intermediate visual outcomes following bilateral implantation, with Clareon demonstrating non-inferiority to Eyhance in intermediate vision performance. In clinical practice, both IOLs may therefore be considered useful options for patients undergoing cataract surgery who expect good distance vision and some degree of spectacle-independent intermediate visual function. Further studies with larger study groups and longer follow-up are needed to confirm these observations.

## Figures and Tables

**Table 1:** Patient demographics and preoperative and postoperative clinical data. Data are presented as *n* (%) or mean ± Standard Deviation (minimum, maximum), except target refraction/aim, which is presented as mean (range).

Characteristic	Clareon Group (*n* = 42 Patients, 84 Eyes)	Eyhance Group (*n* = 44 Patients, 88 Eyes)
Age, y	67.56 ± 6.61 (52, 81)	64.46 ± 6.61 (51, 79)
Gender, *n* (%)		
Female	24 (57.1)	24 (54.5)
Male	18 (42.9)	20 (45.5)
Anterior Chamber Depth (ACD), mm	3.14 ± 0.46 (2.08, 4.53)	3.04 ± 0.48 (2.01, 4.46)
Axial Length (AL), mm	23.11 ± 1.22 (21.28, 26.34)	22.81 ± 1.32 (21.08, 26.24)
Pupil size, mm	3.32 ± 0.92 (1.91, 6.47)	3.12 ± 0.82 (1.88, 6.23)
Corneal Astigmatism (K AST), D	0.61 ± 0.29 (0.00, 1.99)	0.59 ± 0.27 (0.00, 1.99)
Average Corneal Power (average of K1 and K2), D	43.44 ± 1.44 (40.32, 47.11)	43.57 ± 1.47 (40.22, 46.91)
Intraocular Lens Power, D	21.32 ± 2.76 (13.0, 30.0)	21.25 ± 2.69 (15.0, 30.0)
Target Refraction, D; mean range)	−0.31 (−0.5, 0.00)	−0.28 (−0.5, 0.00)
Toric Intraocular Lens, *n* (%)	0 (0.00)	0 (0.00)
Postoperative Sphere, D	−0.26 ± 0.41 (−0.75, 1.25)	−0.29 ± 0.39 (−0.50, 1.25)
Postoperative Residual Astigmatism (PRA), D	0.46 ± 0.52 (0, 1.75)	0.48 ± 0.42 (0, 1.75)
Mean Residual Spherical Equivalent (MRSE), D	−0.27 ± 0.42 (−0.77, 1.49)	−0.29 ± 0.37 (−0.74, 1.52)

**Table 2:** Postoperative visual acuities.

Binocular Visual Acuity	Group	Mean ± SD (Minimum, Maximum) logMAR	95% CI for Difference in Mean, Clareon − Eyhance	*p*-Value
Distance Corrected Intermediate Visual Acuity (DCIVA)	Clareon	0.23 ± 0.09 (0.00, 0.60)	−0.02 to 0.06	0.28
	Eyhance	0.21 ± 0.08 (0.00, 0.51)		
Corrected Distance Visual Acuity (CDVA)	Clareon	0.02 ± 0.02 (0.00, 0.10)	−0.01 to 0.01	1.00
	Eyhance	0.02 ± 0.03 (0.00, 0.10)		

Abbreviations: CI, confidence interval; SD, standard deviation.

## Data Availability

The data supporting the findings of this study are available from the corresponding author upon reasonable request. The data are not publicly available due to privacy and ethical restrictions.
